# Trends in female genital mutilation/cutting in Senegal: what can we learn from successive household surveys in sub-Saharan African countries?

**DOI:** 10.1186/s12939-018-0907-9

**Published:** 2019-01-30

**Authors:** Ngianga-Bakwin Kandala, Bettina Shell-Duncan

**Affiliations:** 1 Department of Mathematics, Physics and Electrical Engineering, Faculty of Engineering and Environment, Northumbria University, Newcastle upon Tyne, NE1 8ST UK; 2 Division of Epidemiology and Biostatistics, School of Public Health, University of the Witwatersrand, Johannesburg, South Africa; 3 Department of Anthropology, University of Washington, Box 353100, Seattle, WA 98195-3100 USA

## Abstract

**Background:**

Over the last several decades, global efforts to end female genital mutilation/cutting (FGM/C) have intensified through combined efforts of international and non-governmental organizations, governments, and religious and civil society groups. One question asked by donors, program implementers and observers alike is whether there is any evidence that FGM/C is declining. In the last two decades, reliable data have been generated in numerous countries through major household surveys, including repeat cross-sectional surveys. What can we learn from these data? We explore this question by analyzing data on FGM/C obtained from women aged 15–49 in two successive household surveys in Senegal (2005 and 2010–11). The aggregate national-level statistics suggest that there has been no significant change in the prevalence of FGM/C among adult women. These figures are, however, unadjusted for potentially confounding factors, and potentially mask important variation in the practice.

This paper aims to provide a deeper understanding of trends in FGM/C across regions, and possibly across generations, providing evidence as to when and where the practice of FGM/C is changing. We aim to answer the following questions: 1. What are the trends in FGM/C among women across Senegal and within regions? 2. Are individual characteristics, such as education, wealth and ethnicity, associated with a likelihood of FGM/C? 3. Are community-level factors, captured by covariate-adjusted geographic estimates, important predictors of a likelihood of FGM/C, as predicted by social convention theory?4. After adjusting for individual- and community-level factors, do we see a decrease in the prevalence of FGM/C across generations of women in Senegal?

**Methods:**

Participants were 14,602 and 14,228 respondents from two consecutive Senegal Demographic and Health Surveys from 2005 to 2010 (FGM/C prevalence 30.1% in 2005 and 28.1% in 2010). A Bayesian geo-additive mixed model based on Markov Chain Monte Carlo techniques was used to map the change in the spatial distribution of FGM/C prevalence at the regional level during the five-year period, while simultaneously examining the effect of individual-level risk factors.

**Results:**

Overall, the prevalence of FGM/C at that national level changed little over the 5-year period, but the fully-adjusted model and map of trends in residual spatial effects at the regional level reveal important spatial patterns. Across both survey periods, several high prevalence regions remained “hot spots,” bearing a consistently high FGM/C prevalence. These include Kolda (along with the newly subdivided region of Sédhiou in 2010), Tambacounda (along with the newly subdivided region of Kédougou in 2010), and Matam. At the same time, risk remained not significant in the high prevalence regions of Saint Louis and Zinguinchor and was attenuated between 2005 and 2010–11 in Kaolack (including the newly subdivided region of Kaffrine in 2010), shifting from not significant risk in 2005 to a very low FGM/C prevalence in 2010–11. In both surveys, unadjusted estimates of the effect of age show no significant difference in risk of FGM across age cohorts. However, non-parametric covariate-adjusted estimates show that in both surveys age is a significant risk factor for FGM/C, although not in the anticipated direction. The effect of age on prevalence of FGM/C is highest in women aged 15–20, and declines with increasing age. Other significant factors are socio-demographic variables, particularly ethnicity.

**Conclusions:**

Findings from two consecutive surveys reveal that while no significant changes in FGM/C prevalence are found at the national level, mixed changes are visible at the regional level, as well as at the individual level. The modelled covariate results confirmed that the patterns of FGM/C differ markedly with region of residence and age remaining significant risk factors in both surveys, suggesting that community factors (convention theory), above and beyond individual factors, play a crucial role in the perpetuation, spread or decline of the practice of FGM/C.

There is a clear pattern of regions with higher prevalence of FGM/C, mostly the south-eastern region of Tambacounda, Kolda and Matam in 2005, including the eastern region of Kédougou and the southern region of Sédhiou in 2010, which were associated with a higher prevalence of FGM/C, while regions  such as Louga, Thiès, Diourbel, Kaolack and Fatick in 2005 and Louga, Thiès, Diourbel, Fatick, Kaolack and Kaffrine in 2010 were associated with a lower prevalence of FGM/C.

However, the total spatial residuals in both surveys also indicate that much of the variation in FGM/C likelihood remains to be explained. The spatial effects of the Kaolack region in 2005 was greatly attenuated after multiple adjustments of other risk factors indicating that perhaps the higher number of FGM/C affected women living in the region was inflated by other factors such as ethnicity, socio-economic status and education. Overall, the results indicate that across surveys, certain high prevalence regions remain “hot spots” regarding FGM/C prevalence.

These novel findings fit with predictions of theory on social norms and conventions which suggest that the practice is upheld by interdependent expectations regarding the practice, and when such expectations are challenged within a community, the possibility for abandonment is opened.

## Introduction

Over the last four decades, global efforts to end female genital mutilation/cutting (FGM/C) have intensified through combined efforts of international and non-governmental organizations, governments, religious and civil society groups. In an effort to track progress, reliable data have been generated in numerous countries through major household surveys, and in some countries repeat surveys have been implemented [1, 2]. What can we learn from these data? We explore this question by analyzing data from two successive household surveys in Senegal (2005 and 2010–11 Demographic and Health Surveys), adopting a novel Bayesian geo-additive model and mapping changes in FGM/C at the regional level. We explore the effects of geographic location, as well as individual factors, in an effort to gain a deeper understanding of trends in female genital mutilation/cutting (FGM/C) across the generations in Senegal.

FGM/C refers to a set of practices altering the female genitalia for non-medical reasons. These range from nicking the tissue surrounding the clitoris to the complete removal of the external genitalia. WHO [3] has classified different types of FGM/C as follows: Type I (clitoridectomy) involves removal of all or part of the clitoris and/or the prepuce; Type II (excision) involves removal of the clitoris and the labia minora; Type III (infibulation) involves removal of all of the external genitalia, and appositioning the labia to form a seal, leaving a pinhole opening for the passage of urine and blood; and Type IV, all unclassified forms, including nicking or symbolic circumcision. UNICEF [4] estimates that worldwide more than two million women have undergone some form of FGM/C, and approximately 3.3 million girls are cut each year.

In Senegal a wide range of intervention strategies have been implemented with the goal of accelerating abandonment of FGM/C. The most well-known and systematically implemented approach has been the holistic community education program developed by the Senegal-based nongovernmental organization Tostan. This program aims to empower communities to participate in their own social, economic, political and cultural development [5]. Their two-year education program focuses on hygiene, problem solving, women’s health and human rights, and culminates in a public declaration to abandon FGM/C [6]. The Tostan program organized between 1998 and 1999 has the abandonment of the practice of FGM/C in Senegal as one of its cardinal points. The program covers a total of twelve villages with 10 villages grouped into Type A (villages that had received Tostan programme and declared publicly to stop FGM/C) and 2 villages classified as Type B (villages that had not previously received the Tostan programme but participated in a public declaration to stop FGM/C), see, Diop, Moreau and Benga [7]. There were a total of 150 individual interviews conducted for women from among the two groups. By 2010, more than 4000 communities in Senegal had participated in public declarations (www.tostan.org, accessed July 2017). Media coverage of these declarations have generated triumphalist stories featuring headlines such as “Victory in sight for revolution over female genital mutilation” [8], leading to speculation about the imminent end of FGM/C.

The Tostan program has also garnered recognition owing to its alignment with a prominent theory of change with respect to FGM/C. Tostan’s focus on coordinating change within communities by organizing public declarations to abandon FGM/C corresponds closely to social convention theory, which has now become the dominant theoretical framework for programs focusing on FGM/C prevention. Social convention theory uses a game-theoretic approach to explain how certain social practices, such as FGM/C, can become locked in place, and most effectively changed through a process of collective action [9, 10]. It highlights that independent behavior change among individuals is difficult, even if they have become opposed to the practice, because reciprocal expectations and sanctions from violating social norms make it costly for an individual or family to opt out [11]. Communicating changed social norms and expectations, and coordinating behavior change among interconnected social actors allows a critical mass of individuals to move to a new equilibrium, and alter behavior without experiencing negative repercussions. For years, the social norms approach and its emphasis on coordinating incentives has come to form the cornerstone of a number of development programs focused on the prevention of FGM/C in Senegal, as well as elsewhere in Africa [1, 12, 13]. This has led many observers to wonder whether in Senegal there has been change in the prevalence of FGM/C detectable in national survey data.

There are two important considerations that guide the development of an appropriate analytical approach for analysing factors associated with FGM/C in currently available nationally-representative data sets. First, the DHS employs a multistage sampling strategy that involves cluster sampling to draw upon women responders; this creates an analytical challenge because observational units are not independent. Hence, statistical analyses that rely on the assumption of independence, such as standard probit and logistic models, are no longer valid. Second, knowledge of nonlinear effects for some covariates means that it is not possible to assume strictly linear predictors. Analytical approaches have been developed to handle each of these issues. Hayford’s [14] analysis of the 1998 Kenya DHS employed hierarchical models, also known as multilevel models, to separately estimate the effect of community-level and individual-level effects on risk of FGM/C. While this approach provides unbiased estimates when individual-level observations are not independent, it does not address the structured spatial effects that arise from cluster sampling. The DHS clusters often include more than one village that are close to each other and share common risk factors, and consequently, the assumption of independence at the geographical level, such as province or region, is not correct. The independence assumption has an inherent problem of consistency: if the location of the event matters, it makes sense to assume that areas close to each other are more similar than those far apart. Therefore, in this study we adopt methods that account for spatial effects on risk of FGM/C that were applied by Kandala et al. [15] in their analysis of the 2003 Nigeria DHS. This novel approach involves using geo-additive, semi-parametric models that control for spatial dependence and possible nonlinear and time-varying covariates. Specifically, we investigate the following questions:

1. What are the trends in FGM/C among women across Senegal and within regions?
2. Are individual characteristics, such as education, wealth and ethnicity, associated with a likelihood of FGM/C?
3. Are community-level factors, captured by covariate-adjusted geographic estimates, important predictors of a likelihood of FGM/C, as predicted by social convention theory?
4. After adjusting for individual- and community-level factors, do we see a decrease in the prevalence of FGM/C across generations of women in Senegal?

## Female genital mutilation/cutting in Senegal

**Fig. 1:**
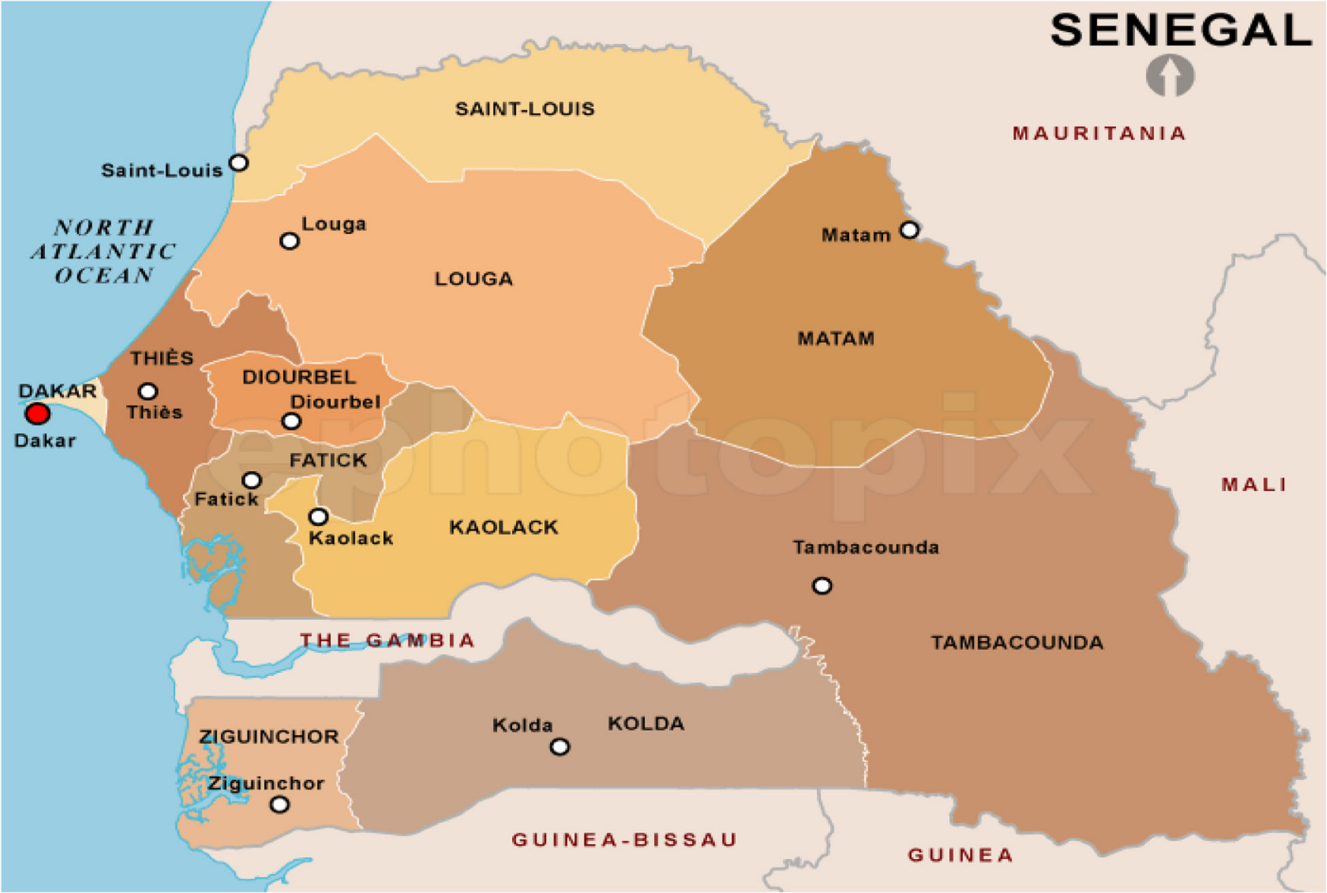
Political map of Senegal showing 11 administrative region  (source: https://www.bing.com)

The Republic of Senegal, with a 2010 population of more than 12.5 million, is home to more than 20 ethnic groups, each with their own language, culture and history. The country is divided into 11 administrative regions **(**Fig. 1**)**. Three additional regions were newly created in 2008, when Kaffrine Region was split from Kaolack, Kédougou was split from Tambacounda, and Sédhiou Region was split from Kolda.

## Methods

### Data source

The data analyzed in this study are from two nationally-representative household surveys: the 2005 Senegal Demographic and Health Survey (SDHS 2005), and the 2010–11 DHS survey with questions from the Multiple Indicator Survey (SDHS-MICS 2010–11). The Demographic Health Surveys (DHS) are periodic cross sectional health surveys funded by USAID (the U.S. Agency for International Development’s) Bureau for Global Health. The DHS includes a number of modules on demographics and household affluence; fertility, reproductive health, maternal and child health, nutrition and knowledge and practice related to HIV/AIDS. The core questionnaire for households collects data from adult women (age 15–49) and men from a nationally representative probability sample of households. Surveys allow for an optional additional series of questions about FGM/C to be added to the women’s questionnaire [16, 17]. The module on FGM/C includes 3 sections: 1) questions on whether the woman underwent FGM/C or not, and details about that event, 2) whether one daughter underwent FGM/C or not, and details about that event, and 3) a woman’s opinion about the continuation of the practice. Since 2000, UNICEF’s Multiple Indicator Cluster Surveys (MICS) have used a similar module to collect information on FGM/C in selected countries [16, 17]. We draw on data from the core questionnaire for households, as well as the module on FGM/C, administered to women age 15–49 years. For the 2010–11 survey, the FGM/C module was modified to ask questions about the FGM/C status of each daughter under the age of 10, rather than about only one daughter, thus providing more detailed information about recent changes in the practice. We are currently undertaking the analyses of daughter data, which is forthcoming and will be presented elsewhere. Further details on the methods, objectives, organization, sample design, and questionnaires used in the 2005 SDHS and 2010–11 SDHS-MICS are described elsewhere [16, 17]. The study protocol conforms to the ethical guidelines of the 1975 Declaration of Helsinki as reflected in a priori approval by the institution’s human research committee. Ethical approval was granted by the Ethics Committee of the National Statistical Office of Senegal.

The sampling strategy for each survey was designed to be nationally representative, and provide information for each region. A two stage sampling process was employed. In the first stage clusters (377 in 2005, and 391 in 2010–11) were selected from a list of enumeration areas with probability proportional to size. In the second stage, a complete household listing was completed in each selected cluster, followed by the random selection of 21 households per cluster. In each household, all women age 15–49 were interviewed. In 2005 survey data were collected from a total of 7412 households and 14,602 women aged 15–49, with a response rate of 93.7%. The 2010–11 survey selected 8211 households, and collected data from 15,688 women aged 15–49, with a response rate of 92.7%. Further details on sampling can be found elsewhere [18]. For both surveys, there were few participants with missing data for FGM/C and other covariates; thus, data analysis on FGM/C was based on 14,602 women in 2005 and 14,228 women in 2010–11 with a complete set of data.

### Outcome variable

We studied FGM/C as the main outcome in terms of “whether a participant had had FGM/C performed on her”. This question was converted into a binary variable, with two categories defined as 1 if the participant was cut and 0 if the participant had no FGM/C performed on her.

### Exposure variables

The main exposure variable in the analysis was the “region of residence” (of which there are 11 in 2005 as shown in Figs. 1, and 14 in 2010–11), in addition to various control variables on socio-demographic factors potentially associated with FGM/C: sex, age, education level, wealth index, marital status, family size, place of residence (urban vs. rural) (See Table 2).

## Statistical analysis

The 2005 to 2010–11 of the Senegalese DHS include geographical information that allow us to examine spatial effects along with individual sociodemographic factors that may influence the risk of FGM/C. These factors are explored within a simultaneous, coherent regression framework, using a geo-additive, semi-parametric mixed model that simultaneously controls spatial dependence and possibly nonlinear or time effects of covariates and the complex sampling design [15].

Historically, variations in prevalence of FGM/C have been related to household socio-economic factors (such as education, age, income). However, geographical associations with prevalence have been neglected. In this paper, we begin with a simple analysis of geographical variation of FGM/C in Senegal followed by a more detailed approach to the data.

First, a conventional logistic regression analysis was carried out to document regions’ differences in the variations of the observed FGM/C rates using dummies variables for the 11 and 14 regions from the 2005 and 2010–11 surveys, respectively. A map of observed FGM/C prevalence was produced from the raw data. We also tested the bivariate and multivariate associations of well-known socioeconomic correlates of FGM/C. From this initial analysis, we identified the association between socio-economic and demographic factors and FGM/C while showing the regions’ differences in the prevalence of FGM/C and the variation across them in the correlates (results not shown here).

Second, we then used flexible methods to model spatial determinants of FGM/C and to allocate these spatial effects to structured and unstructured (random) components. This modelling draws on Bayesian geo-additive methods of spatial statistics, taking advantage of advances in Geographic Information Systems [15, 19]. The modelling of the two components is done jointly in one estimation procedure that thereby simultaneously identifies socioeconomic determinants, and the spatial effects that are not explained by these socioeconomic determinants while accounting for the complex sampling scheme. In this way, we are able to identify regional patterns of FGM/C that are either related to left-out socioeconomic variables that have a clear spatial pattern or point to spatial (possibly cultural or environmental) processes that account for these spatial patterns.

### Data structures

Nested data in survey studies is often the rule rather than the exception. Here the data structure is retrospective birth, health and survival information including self-reported history of the FGM/C practice, typically about more than one child from each sampled woman. Health and survival information of women and their children are nested within family, the clustering of families living within regions. In fact, heterogeneity is often present and frequently the available predictor variables do not explain this heterogeneity sufficiently, see [20, 21]. With recent computational advances in statistics it is becoming increasingly straightforward to describe such heterogeneity with mixture models that employ unobserved predictors in a Bayesian hierarchical structure.

### Sample design

Administratively, Senegal was divided in 11 administrative regions in 2002 and regions were subdivided into 34 departments, which in turn included 66 urban communes, 94 arrondissements and 320 rural communities.

The sample is drawn through stratified clustered sampling and draws, in the case of the 2005 SDHS, 377 clusters (158 urban areas and 219 in rural areas) from 34 departments from 11 regions. In total, 8000 households were drawn at random to select a representative national sample of 12,000 women aged 15–49 and 3400 men aged 15–59. The response rates were 93.7% for women and 86% for eligible men. However, one cannot assume that the clusters selected in each region are fully representative of the region in which they are located, as the surveys only attempted to generate a fully representative sample at the national level. Consequently, the spatial analysis will be affected by some random fluctuations. Some of this random variation can be reduced through the relaxation of the independence assumption between neighbouring states. Such a spatial analysis should preferably be applied to census data, where there is higher clustering at the highly disaggregated sub-national level and the precision of the spatial analysis would be much higher. Unfortunately, most censuses do not collect data on FGM/C and often the full dataset is not available for such analyses. Hence, analysis of the household survey data provided by the SDHS is the only feasible way to evaluate spatial variation of FGM/C.

We assess the likely impact of the neglect of hierarchical structure and geographical location in analyses of the SDHS data that ignore correlation structure and dependence in the data. The neglect of the geographical location where the respondent lives leads to underestimation of standard errors of the fixed effects that inflates the apparent significance of the estimates [15, 22]. Our analysis includes this correlation structure and accounts for the dependence of neighbouring communities (regions) in the model. The model also permits ‘borrowing strength’ from neighbouring areas to obtain estimates for areas that may, on their own, have inadequate sample sizes. This gives more reliable estimates of the fixed effect standard error. More detailed information about the formulated models is presented in the appendix.

## Results

### Descriptive results

The data summarized in Table 1 describes the circumstances surrounding the practice of FGM/C in Senegal. FGM/C typically occurs at very young ages, with the majority of girls cut by age 1, and over 70% cut by age 4. The most common form of cutting is “cut/flesh removed,” which may correspond to FGM Type I (clitoridectomy) or Type II (excision).^1^ In contrast to other parts of Africa such as Egypt, Kenya and Sudan, FGM/C in Senegal has not become medicalized (performed by health professionals). Support for the continuation of FGM/C (18% in 2005 and 17% in 2010–11) is lower than the estimated national prevalence of FGM/C (weighted estimates are 28% in 2005 and 26% in 2010) [1]. Overall, aggregate statistics on prevalence, unadjusted for potentially confounding factors, provide the impression that there is very little change in FGM/C among women in Senegal. In this paper we present results from multivariate analyses that highlight patterns in the data after adjusting for the effect of proximate variables. Specifically, using data from successive household surveys, we examine the spatial distribution of FGM/C, and estimate the effects of a number of sociodemographic factors that could mediate the observed prevalence of FGM/C in Senegal.

**Table 1:** Circumstances Surrounding FGM/C in Senegal

	SDHS 20005	SDHS 2010–11
Age at cutting (%, cumulative %)		
0–1	63.3	61.7
2–4	9.5 (72.8)	9.5 (71.2)
5–9	14.9 (87.7)	13.8 (85.0)
10–14	5.1 (92.8)	6.0 (91.0)
15+	0.9 (93.7)	0.7 (91.7)
Type of FGM/C (%)		
Cut, no flesh removed	0	10
Cut, flesh removed	83	53
Sewn closed	12	14
Not sure/don’t know	5	24
Practitioner of FGM/C (%)		
Traditional practitioner	93	100
Medical practitioner	1	0
Don’t know	7	0
Support continuation (all women, both cut and uncut) (%)	53	52
Prevalence (weighted %)	28	26

Source: [1]; [2]; [16]

Unweighted baseline socio-demographic characteristics are shown in Table 1**,** and by FGM/C status (whether a participant underwent FGM/C or not) in Table 2. The overall prevalence of FGM/C differs slightly between the two surveys (30.1% in 2005 and 28.1% in 2010–11 unweighted). Before investigating factors associated with FGM/C and trends across the two surveys, we examined comparability of women sampled in each survey. The two survey populations are similar in terms of the mean ages of women (for 2005 the mean age was 27.8 years, with a standard deviation of ±9.9 years and in 2010–11 the mean age of the sample was 27.9 years, with a standard deviation of ±9.5 years). Most of the population sampled lived in rural settings (51.3% in 2005 and 50.7% in 2010–11) and 73.0% were married in 2005 with slightly less at 66.5% in 2010–11. The mean age for women’s partner was higher than that for women in both the 2005 (42.6 vs. 27.8 years) and the 2010–11 survey (43.5 vs. 27.9 years). A total of 14.2% of women in the 2005 population had a secondary education while 59.6% had no education compared to 18.3% (secondary education) and 57.9% no education in 2010–11. Women with FGM/C were mostly married (34.0% vs 30.6%), with no education for themselves (34.4% vs 31.4%) or their partners (36.7% vs 32.2%), were “poorest” (43.8% vs 48.7%), had a large family size (41.0% vs 35.5%), lived in rural areas (37.7% vs 35.6%), were Soninke (78.7% vs 66.3%), were Muslim (31.0% vs 29.0%) and were living in Matam and Kédougou (94.9% vs 87.7%) and Kolda and Matam (94.3% vs 89.6%) in 2005 and 2010–11 respectively. Notably, in both surveys, we found that unadjusted estimates of the effect of age show no significant difference in the risk of FGM/C across the age cohorts in both surveys (2005 and 2010–11). However, the non-parametric covariate-adjusted estimates show that the effect of age on the risk of FGM/C was at its peak among women aged 15–20, and decreases with increasing age. It was found that place of residence (urban/rural), ethnicity, religion and region of residence were factors responsible for this unanticipated change.

**Table 2:** Baseline characteristics of the study population of women ages 15–49 (Senegal DHS, 2005 & 2010)^a^

Variable	Women 2005 (*N* = 14,602)	Women 2010 (*N* = 15,688)
Mean age^b^ (SD) respondent	27.8 (9.9)	27.9 (9.5)
Mean age^b^ (SD) partner	42.6 (9.9)	43.5 (17.5)
FGM/C (%)		
Yes	30.1	28.1
No	69.9	71.9
Place of residence (%)		
Urban	48.7	49.3
Rural	51.3	50.7
Married (%)		
Yes	73.0	66.5
No	27.0	33.5
Education (%)		
No education	59.6	57.9
Primary education	25.2	21.8
Secondary education	14.2	18.3
Higher education	1.0	2.1
Partner’s education (%)		
No education	70.3	75.4
Primary education	12.6	11.9
Secondary education	12.9	9.2
Higher education	4.3	3.5
Wealth Index (%)		
Poorest	16.7	16.5
Poorer	17.6	17.9
Middle	19.4	19.8
Richer	21.6	22.3
Richest	24.7	23.5
Family size (%)		
Small (1–4 children)	84.6	82.1
Middle (5–7 children)	13.0	15.4
Large (8+ children)	2.5	2.6
Ethnicity (%)		
Wolof	39.7	38.7
Poular	25.2	26.5
Serer	15.9	15.0
Mandingu	4.6	4.2
Diola	4.9	4.0
Soninke	2.8	2.3
Not Senegalese	1.7	2.0
Other	5.2	7.3
Religion (%)		
Muslim	95.5	95.4
Other	4.5	4.6
Region of residence (%)		
Dakar	26.5	26.0
Diourbel	10.6	11.8
Fatick	4.8	4.6
Kaffrine		3.7
Kaolack	11.3	7.5
Kédougou		0.7
Kolda	7.2	4.1
Louga	6.3	7.2
Matam	3.7	3.8
Saint Louis	6.5	6.6
Sédhiou		2.9
Tambacounda	5.8	4.6
Thiès	13.5	12.9
Ziguinch	3.9	3.7

^a^Data are expressed as mean (standard deviation) or as percentages

^b^Age ranges from 15 to 49 year for women, and 18 to 97 years for partners

It is important to point out that although the prevalence of FGM/C between the two successive surveys was not statistically significantly different (30.1% in 2005 and 28.1% in 2010-11) and in terms of statistical modelling strategy, the surveys were modelled separately rather than jointly with a time dummy and exploration of other time interactions because our important research question was to investigate the magnitude and change of the spatial effects at the regional level. In the exploratory analysis, we noticed that they were higher FGM/C prevalence regions in the 2005 survey that reduced significantly their FGM/C prevalence in 2010. Such regions were Kaolack and Kolda. Therefore, we modelled the two surveys separately to investigate this shuttle change in prevalence as the two surveys have not been analyzed to investigate FGM/C separately in the past.

### Regression analysis results

Unadjusted odds ratios (ORs) and fully adjusted posterior odds ratios (PORs) are presented in Table 3. In 2005 factors associated with FGM/C in the unadjusted analysis were: rural place of residence (OR = 2.09, 95% CI = 1.94–2.25), being married (OR = 2.22, 95% CI = 1.99–2.47), with “no education” (OR = 2.05, 95% CI = 1.13–3.70), wealth index with poorer quintile in the lead (OR = 5.37, 95% CI = 4.59–6.29), being Soninke (OR = 3.70, 95% CI = 2.62–5.21) or Mandingu (OR = 2.96, 95% CI = 2.36–3.72), being Muslim (OR = 3.54, 95% CI = 2.70–4.64) and living in Matam (OR = 18.2, 95% CI = 14.0–23.7) or Kolda (OR = 16.5, 95% CI = 13.2–20.6). After adjusting for all other factors (fully adjusted model), the likelihood of FGM/C in women with only primary or secondary education became not statistically significant. The effect disappeared also for small and middle family size; the statistically significant effect remains only for women living in rural areas, with no education, partners with primary education, in all wealth quintiles, all ethnicities, and the region of residence. Women with no education were 3.26 times more likely to undergo FGM/C than all higher educated persons (95% CI = 1.04–10.1); women of the poorest wealth quintile were 4.60 times more likely to be cut than those from the richest; women living in rural communities were 1.47 times more likely to have undergone FGM/C (95% CI = 1.31–1.65) than women living in urban areas, and 3.02 times more likely to be Muslim than another religion; Soninke women were 75.4 times more likely to undergo FGM/C than Wolof women. Women with FGM/C were least likely to live in Louga and Fatick and most likely in Matam and Kolda.

**Table 3:** Unadjusted and fully adjusted odds ratios of women’s circumcision across selected covariates (Senegal, DHS 2005 & 2010)

Variable	Women 2005		Women 2010-11	
	Unadjusted OR & 95%CI^a^	Fully adjusted POR & 95% CI^b^	Unadjusted OR & 95%CI^a^	Fully adjusted POR & 95% CI^b^
Age				
15–19 years	0.93(0.81, 1.07)		1.00(0.86,1.15)	
20–24 years	1.00(0.86, 1.15)	See Fig. 4 left	0.93(0.80,1.08)	
25–29 years	1.00		1.00	
30–34 years	1.08(0.92, 1.27)		0.94(0.80,1.10)	
35–39 years	1.08(0.91, 1.27)		1.12(0.95, 1.33)	
40–44 years	1.08(0.91, 1.29)		1.02(0.85, 1.23)	
45–49 years	1.09(0.90, 1.32)		1.08(0.88, 1.33)	
Age Partner				
15–30 years	1.00	See Fig. 4 right	1.01(0.88,1.16)	
31–49 years	0.80		1.00	
50–60 years	0.82		0.98(0.86, 1.12)	
> 61 years	1.10		1.28(1.06, 1.54)	
Place of residence				
Urban	1.00	1.00	1.00	1.00
Rural	2.09(1.94, 2.25)	1.47(1.31, 1.65)	1.42(1.32, 1.53)	0.78(0.70, 0.87)
Married				
Yes	2.22(1.99, 2.47)		1.47(1.33, 1.63)	
No	1.00		1.00	
Education				
No education	2.05(1.13, 3.70)	3.26(1.04, 10.1)	4.10(2.57, 6.53)	1.86(0.74, 4.65)
Primary education	1.42(0.78, 2.57)	3.11(0.99, 9.73)	3.55(2.21, 5.68)	1.77(0.71, 4.45)
Secondary educ.	0.93(0.51, 1.71)	2.41(0.76, 7.63)	2.87(1.79, 4.61)	1.62(0.64, 4.13)
Higher education	1.00	1.00	1.00	1.00
Partner’s education				
No education	1.96(1.44, 2.68)	1.30(0.87, 1.93)	1.69(1.18, 2.43)	1.21(0.81, 1.80)
Primary education	1.58(1.12, 2.22)	1.58(1.12, 2.22)	1.55(1.04, 2.30)	1.45(0.95, 2.21)
Secondary educ.	1.33(0.94, 1.88)	1.33(0.94, 1.88)	1.48(0.98, 2.23)	1.43(0.92, 2.22)
Higher education	1.00	1.00	1.00	1.00
Wealth Index				
Poorest	5.03(4.30, 5.88)	4.60(3.61, 5.86)	5.21(4.42, 6.14)	5.77(4.55, 7.33)
Poorer	5.37(4.59, 6.29)	4.52(3.57, 5.72)	3.05(2.58, 3.60)	3.35(2.64, 4.27)
Middle	3.42(2.92, 4.02)	2.44(1.94, 3.07)	2.22(1.87, 2.64)	2.16(1.70, 2.73)
Richer	1.97(1.64, 2.37)	1.79(1.37, 2.33)	1.51(1.25, 1.83)	1.37(1.05, 1.79)
Richest	1.00	1.00	1.00	1.00
Family size				
Small (1–4 children)	0.60(0.50, 0.73)	0.98(0.79, 1.23)	1.00	1.00
Middle (5-7children)	0.66(0.53, 0.83)	0.90(0.70, 1.16)	1.22(1.08, 1.37)	1.16(1.00, 1.33)
Large (8+ children)	1.00	1.00	1.46(1.19, 1.80)	1.11(0.88, 1.42)
Ethnicity				
Wolof	1.00	1.00	1.00	1.00
Poular	1.79(1.67, 1.93)	23.9(20.1, 28.5)	129(98.6, 169)	19.9(16.9, 23.5)
Serer	0.02(0.01, 0.03)	0.22(0.14, 0.36)	2.56(1.70, 3.85)	0.39(0.26, 0.60)
Mandingu	2.96(2.36, 3.72)	67.3(45.4, 99.6)	478(326, 702)	89.4(55.5, 144)
Diola	1.53(1.26, 1.87)	20.3(14.3, 28.6)	125(89.6, 174)	29.4(20.0, 43.3)
Soninke	3.70(2.62, 5.21)	75.4(44.1, 129)	185(123, 280)	31.2(17.1, 56.6)
Not Senegalese	2.49(1.69, 3.67)	33.9(20.6, 55.9)	168(107, 264)	24.8(15.4, 39.9)
Other	0.64(0.54, 0.75)	11.2(8.41, 14.9)	50.1(36.8, 68.3)	9.22(6.88, 12.3)
Religion				
Muslim	3.54(2.70, 4.64)	3.02(2.11, 4.34)	4.10(2.92, 5.77)	2.52(1.61, 3.96)
Other	1.00	1.00	1.00	1.00
Region of residence				
Dakar	0.22(0.19, 0.25)		0.26(0.23, 0.31)	
Diourbel	1.00		1.00	
Fatick	0.07(0.05, 0.08)		0.11(0.09, 0.13)	
Kaffrine	New state in 2010		0.12(0.10, 0.15)	
Kaolack	0.14(0.12, 0.16)	See Fig. 2	0.08(0.06, 0.10)	
Kédougou	New state in 2010		14.5(10.4, 20.4)	
Kolda	16.5(13.2, 20.6)		7.10(6.02, 8.39)	
Louga	0.05(0.04, 0.07)		0.05(0.04, 0.05)	
Matam	18.2(14.0, 23.7)		8.65(7.12, 10.5)	
Saint Louis	0.88(0.84, 0.93)		0.78(0.72, 0.85)	
Sédhiou	New state in 2010		8.29(6.90, 9.94)	
Tambacounda	6.98(5.95, 8.19)		7.32(6.20, 8.63)	
Thiès	0.08(0.07, 0.10)		0.04(0.03, 0.05)	
Ziguinchor	2.32(2.08, 2.58)		1.59(1.40, 1.80)	

^a^Unadjusted marginal odds ratio (OR) from standard logistic regression models.

^b^Adjusted posterior odds ratio (POR) from Bayesian geo-additive regression models

In the following survey year of 2010–11**,** factors associated with FGM/C in the unadjusted analysis were: rural place of residence (OR = 1.42, 95% CI = 1.32–1.53); being married (OR = 1.47, 95% CI = 1.33–1.63); education, with the category “no education” highest (OR = 4.10, 95% CI = 2.57–6.53), followed by primary education (OR = 3.55, 95% CI = 2.21–5.68) and secondary (OR = 2.87, 95% CI = 1.79–4.61) vs people with higher education; wealth index for women in the poorest quintile (OR = 5.21, 95% CI = 4.42–6.14), poorer women (OR = 3.05, 95% CI = 2.58–3.60), middle income (OR = 2.22, 95% CI = 1.87–2.64), and richer women (OR = 1.51, 95% CI = 1.25–1.83), as compared to richest women; family size for middle size family of 5–7 children (OR = 1.22, 95% CI = 1.08–1.37), and for large family size of 8+ children (OR = 1.46, 95% CI = 1.19–1.80) vs people with a small size family (1–4 children); ethnicity; religion and region of residence. After adjusting for all other factors, the likelihood of FGM/C in women living rural with any education became not statistically significant. The effect disappeared also for middle and large family size and partner’s education. A statistically significant effect remained only for the wealth index, ethnicity and religion. Women from the poorest quintile were 5.77 times more likely to undergo FGM/C than the richest (95% CI = 4.55–7.33), participants from the Mandingu tribe were 89.4 times more likely to be cut than people from the Wolof (95% CI = 55.5–144); women who were Muslim were 2.52 times more likely to have FGM/C than women who were not (95% CI = 1.61–3.96). Women with FGM/C were least likely to be in Thiès and Louga and most likely in Matam and Sédhiou.

Odds ratios of 129 and 478 predicted by the unadjusted models for ethnic groups of Poular and Mandingu respectively indicate a very high likelihood of undergoing FGM/C as compared to the Wolof ethnic group as predicted by the model. In terms of policy, this implies that children of women from the Poular and Mandingu ethnic groups are at higher risk of undergoing FGM compared to their counterparts in the Wolof ethnic group.

### The shift in the prevalence of FGM at the region level and across age cohorts during the 5 years period

Looking at the overall national prevalence of FGM/C between the 5 years period, there was only a slight decrease of 2 percentage points from 30.1% in 2005 to 28.1% in 2010–11 (unweighted average). Aggregate national figures on FGM/C prevalence, however, conceal important spatial variation at the region level within the survey periods. By the time of the 2010–11 survey, 3 regions had been subdivided; to appropriately compare trends in prevalence, we reported the unweighted average of subdivided regions in 2010–11. The observed FGM/C prevalence at the regional level shown in Table 3 indicates that the regions in which FGM/C prevalence is lowest and below the national average in both surveys are Dakar, Diourbel, Fatick, Kaolack (along with Kaffrine in 2010–11), Louga and Thiès. The prevalence of FGM/C was consistently higher than the national average in both surveys in Kolda (combined with Sédhiou in 2010–11), Matam, Saint Louis, Tambacounda (combined with Kédougou in 2010–11). Between 2005 and 2010–11 the prevalence of FGM/C increased slightly in the low prevalence regions of Dakar (18.0 to 20.9%) and Fatick (6.2 to 9.6%). It reduced slightly in the low prevalence regions of Diourbel (2.0 to 0.5%) and Thiès (7.3 to 3.8%). The high prevalence region of Tambacunda saw no substantial change in the prevalence of FGM/C between the 2005 and 2010–11 surveys (87.5 to 88.7), while decreases were seen in Kolda (94.3 to 88.3), Matam (94.8 to 89.6), Saint Louis (46.9 to 44.0), and especially Ziguinchor (69.8 to 61.3). Unadjusted marginal odds ratios shown in Table 3 indicate that in 2005 the highest risk of FGM/C was in Matam (OR = 18.2, 95% CI = 14.0–23.7) and Kolda (OR16.5, 95% CI = 13.2–20.6), and the lowest risk in Louga (OR = 0.05, 95% CI = 0.04–0.07). In 2010–11, the highest risk was in Kédougou, and the lowest was in Thiès and again in Louga. Regarding the effect of age, the unadjusted marginal odds ratios show no significant differences in FGM/C risk across age cohorts of women, suggesting that there is no secular decline in FGM/C risk.

### Bayesian spatial analysis results

**Fig. 2:**
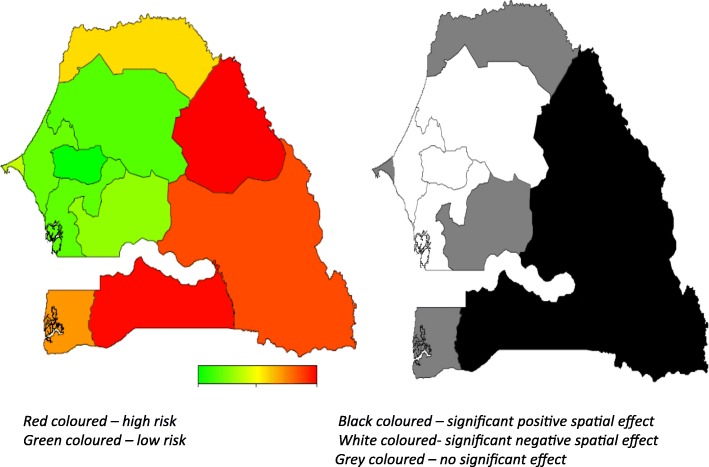
Left: Adjusted total residual spatial effects for women’s circumcision, at regions level in Senegal in 2005. Shown are the posterior odds ratios. Right: Corresponding posterior probabilities at 90% nominal level (SDHS, 2005)

**Fig. 3:**
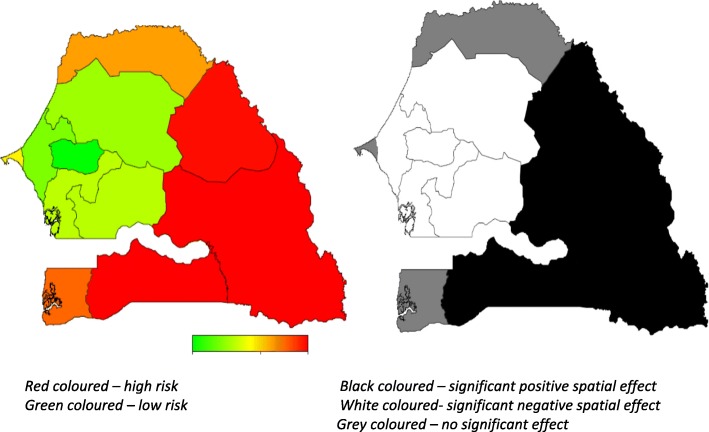
Left: Adjusted total residual spatial effects for women circumcision, at regions level in Senegal in 2010-11. Shown are the posterior odds ratios. Right: Corresponding posterior probabilities at 90% nominal level (SDHS, 2010-11)

**Fig. 4:**
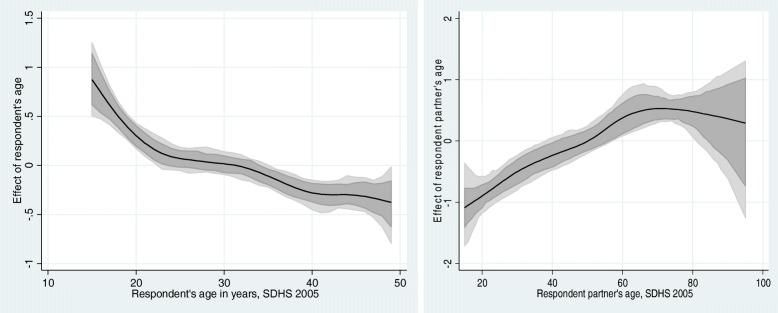
Left: Estimated nonparametric trend of women’s FGM by respondent’s age cohort (left) and respondent partner’s age cohort in 2005 (right). Shown is the posterior mean within 80% credible regions. [SDHS 2005]

**Fig. 5:**
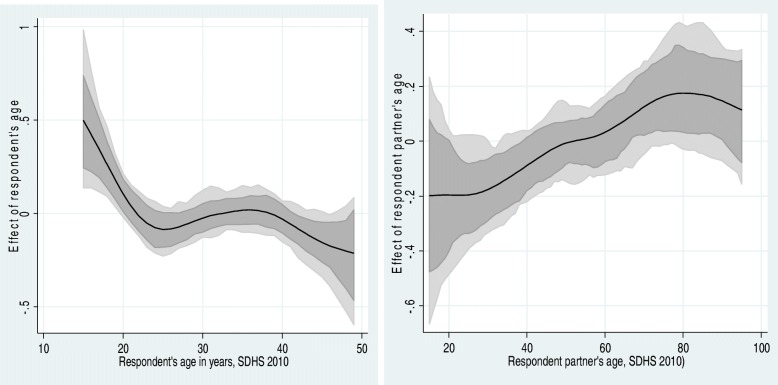
Left: Estimated nonparametric trend of women’s FGM by respondent’s age cohort (left) and respondent partner’s age cohort in 2010-11 (right). Shown is the posterior mean within 80% credible regions. [SDHS 2010-11]

In multivariable Bayesian geo-additive regression analyses, we introduced and controlled for spatial and nonlinear factors associated with FGM/C prevalence in both years. Region of residence was modelled as a spatial variable in Figs. 2 and 3, and age of the respondent at the time of interview was modelled as a continuous variable using a flexible nonlinear curve in Figs. 4 and 5. The modelled covariate results confirmed what was observed in the logistic regression analysis but the patterns differ markedly with region of residence and age remaining a significant risk factors in both surveys. Overall, results of 2005 (Fig. 2) show that after accounting for (i) sampling error in the observed data; (ii) relationships with covariates and the uncertainty in the form of these relationships); (iii) uncertainty in the spatial autocorrelation structure of the outcome variable, the regions with the highest FGM/C likelihood included Tambacounda, Kolda, Matam and Ziguinchor, but not Saint Louis. In 2010=11 (Fig. 3), the highest prevalence regions included Matam, Tambacounda, Kolda, and the newly formed regions of Sédhiou and Kédougou, but again Saint Louis was among the regions with lower prevalence.

With regard to the shift of FGM/C by regions, in both samples, the spatial analysis has captured the substantial variation in FGM/C prevalence across regions observed in the marginal regression analyses. The results shown in Figs. 2 and 3 are covariate-adjusted region FGM/C spatial variation captured by the global total residual region effects (i.e. the sum of the unstructured and structured spatial effects). There is a clear pattern of regions with higher prevalence of FGM/C, mostly the south-eastern states of Tambacounda, Kolda and Matam in 2005, including the eastern state of Kédougou and the southern region of Sédhiou in 2010 (Figs. 4 and 5), were associated with a higher prevalence of FGM/C, while states such as Louga, Thiès, Diourbel, Kaolack and Fatick in 2005 and Louga, Thiès, Diourbel, Fatick, Kaolack and Kaffrine in 2010 were associated with a lower prevalence of FGM/C. These spatial patterns confirm the observed marginal model findings shown in Table 3 with a shift observed for Kaolack region, which moved from a significant FGM/C prevalence area in 2005 to a very low significant FGM/C prevalence in 2010-11 (Kaffrine and Kaolack combined).

Specifically, the left-hand map in Fig. 3 shows estimated posterior total residual odds (POR) of FGM/C for each region in 2005, ranging from a lower POR of 0.04 (0.01, 0.18) in Diourbel to a higher POR of 27.83 (7.36, 132.76) in Kolda and in 2010-11 the POR ranges as low as 0.02(0.00, 0.07) in Diourbel to a higher POR of 17.57(3.96, 67.30) in Matam, with red color indicating the highest prevalence recorded and green color denoting lowest prevalence. The right-hand map shows the 95% posterior probability map of FGM/C, which indicates the statistical significance associated with the total excess risk. White indicates a negative spatial effect (associated with reduced risk of FGM/C prevalence), black a positive effect (an increased risk) and grey a not significant effect. However, the total spatial residuals in Figs. 2 and 3 in both surveys show that much of the variation in FGM/C likelihood remains to be explained. The spatial effects of the Kaolack region in 2005 was greatly attenuated after multiple adjustments of other risk factors indicating that perhaps the higher number of FGM/C affected women living in the state was inflated by other factors such as ethnicity, socio-economic status and education. Overall, the results indicate that across surveys, certain high prevalence regions remain “hot spots” regarding FGM/C prevalence. These include Kolda (along with the newly subdivided region of Sédhiou in 2010), Tambacounda (along with the newly subdivided region of Kédougou in 2010), and Matam. Saint Louis and Zinguinchor remained low prevalence regions while in Kaolack there was a shift to a lower FGM/C prevalence between 2005 and 2010–11.

Figures 4 and 5 show the estimated nonparametric trend of women’s FGM/C risk by respondent’s age cohort (left) and respondent partner’s age cohort in 2005 and 2010-11 (right). Shown is the posterior mean within 80% credible regions. Surprisingly, the figures show an inverse U-shape non-linear relationship between the likelihood of FGM/C and women’s age, with a higher prevalence of FGM/C for younger cohorts (under 20 years of age) for both samples. The nonlinear association between age and the likelihood of FGM/C under age 20 does not differ in the two samples starting very high, with a gradual decrease thereafter in both surveys. At all other ages, the two surveys also show agreement in the pattern of decreasing likelihood of FGM/C until age 49. At age 40, this probability decreases quickly as age increases, although the variation in probability increases rapidly at the same time. For women over age 45, there are wide confidence intervals suggesting few cases of FGM/C in both samples that make it difficult to discern a consistent downward decrease in the FGM/C prevalence for this cohort (instability of the estimates) in both surveys. It is worth mentioning that at first glance, the figures seem to be different. However, a careful examination of the two figures reveals that in both figures the age’s effects start at an estimate of 1, with a gradual decrease thereafter. Thus, contrary to expectations, the adjusted non-parametric estimates reveal that the prevalence of FGM/C increases with decreasing age.

We run a step-wise regression analysis to identify which factors were responsible for the change of age effects. It turns out that, although most factors we included in the final model impacted on the effects of age, factors that have a significant impact or factors confounding the effects of respondent’s age on the likelihood of FGM were the place of residence (urban/rural), the ethnicity, religion and the region of residence.

## Discussion

When tracking changes in the prevalence of FGM/C using nationally-representative survey data, it is important to bear in mind that aggregate figures at the national level may mask important variation along the lines of ethnicity or region. Consequently, it is useful to disaggregate the data, and control for potentially confounding factors [23]. This study has used advanced statistical methodology to analyze survey data collected with complex sampling strategies and including possible non-linear covariates. Importantly, this novel approach, adapted from Kandala et al. [15], makes it possible to simultaneously examine individual-level and spatial variability at the sub-national level. Overall, among women aged 15–49 in Senegal, the prevalence of FGM/C has changed little over the 5 year period between successive surveys carried out in 2005 and 2010–11 (FGM/C prevalence from 30.1% in 2005 to 28.1% in 2010–11. We find that these unadjusted figures do indeed mask important variation at both the regional and individual levels.

The modelled covariate results confirmed that the patterns of FGM/C differ markedly with region of residence and age, which remain significant risk factors in both surveys, suggesting that community factors (convention theory), above and beyond individual factors, play a crucial role in the perpetuation of the practice of FGM/C. We find a clear pattern of regions with higher prevalence of FGM/C, mostly the south-eastern states of Tambacounda, Kolda and Matam in 2005 and the eastern state of Kédougou and the southern region of Sédhiou in 2010 were associated with a higher prevalence of FGM/C, while states such as Louga, Thiès, Diourbel, Kaolack and Fatick in 2005 and Louga, Thiès, Diourbel, Fatick, Kaolack and Kaffrine in 2010 were associated with a lower prevalence of FGM/C.

In two other high prevalence regions- Saint Louis and Zinguinchor- risk of FGM/C remained not statistically significant across both surveys. Additionally, the likelihood of FGM/C was attenuated between 2005 and 2010–11 in Kaolack (including the newly subdivided region of Kaffrine in 2010–11), shifting from being not significant in 2005 to very low risk of FGM/C in 2010–11. It should be noted that in the multivariate Bayesian geo-additive regression analysis we controlled for individual-level and community factors while simultaneously modelling the region of residence as a spatial variable.

In both survey years, 'no education' has an impact on the likelihood of FGM for both a woman and her partner as 'no education' was associated with a higher likelihood of FGM in the unadjusted analysis in both years. However, when we controlled for many other confounding factors in the multivariate analysis, the relationship disappeared and became statistically insignificant. Despite this mixed result, it is important to highlight the role of education at the individual women/partner level as shown by the significant results of the observed unadjusted analysis. However, above the positive impact of education at the individual level, community factors such as the region of residence play a key role in the prevalence of FGM as shown by the significant results of the spatial covariate adjusted multivariate model.

How can we interpret these spatial findings given that certain high prevalence regions remained “hot spots” regarding FGM/C risk and others did not? Importantly, these results show that community-level effects, above and beyond individual-level effects, play a crucial role in determining the likelihood of FGM/C. In other words, the context in which an individual woman lives bears an important influence on whether FGM/C is practiced [23]. This finding is consistent with the social convention theory, which predicts that interdependent expectations and social norms shared by community members serve to uphold the practice, making it difficult for individuals to abandon FGM/C without experiencing adverse social sanctions [11]. The theory predicts that change is most likely to come about when members of social groups have simultaneously shifted social norms pertaining to FGM/C [10]. It may be the case that regional differences in FGM/C risk capture this shift in social norms. At the same time, theory on social norms and conventions does not rule out the possibility of individual-level factors influencing decision-making regarding FGM/C, although the social environment can constrain these choices. Indeed, in our study we find evidence for the simultaneous influence of community- and individual-level factors influencing the risk of FGM/C.

In the fully adjusted model, a number of individual-level factors were found to be associated with the likelihood of FGM/C. In the 2005 survey data these include rural residence, no education, wealth, religion, and especially ethnicity (Soninke women were 75.4 times more likely to be cut than Wolof women) and age. In 2010–11, significant individual-level variables in the fully adjusted model included wealth, religion, and especially ethnicity (Mankingu women were 89.4 times more likely to be cut than Wolof women) and age. The consistent and robust effect of ethnicity is unsurprising given that in Senegal, like many other settings, FGM/C derives much of its meaning and tenacity from its intimate association with ethnic identity [24]. Where a strong link between FGM/C and ethnicity exists, as is the case in Senegal, ethnicity may serve to signal shared expectations that hold the practice in place. In other words, ethnicity may be a proxy for shared norms concerning personhood, religion, sexual restraint or other cultural values. Hence, it is increasingly understood that programmes should be uniquely tailored to address these issues [1].

Our most surprising finding with respect to individual-level predictors of risk of FGM/C among women in Senegal is with respect to age. Unadjusted estimates of risk of FGM/C showed no significant variation across age cohorts. However, fully adjusted nonparametric estimates show that in both surveys, age is a significant risk factor for FGM/C, but not in the anticipated direction. The effect of age on the likelihood of FGM/C is highest in women aged 15–20, and declines with increasing age. How can we understand this unexpected finding? This finding draws a support from a recent work by one of the co-authors [25] to be published in PLoS ONE, in which it was found that younger women are more resistant to change because they have the most to learn in terms of losing social support in their marital home. They depend on older women for support, advice and assistance of all kinds. Older women were more open to change because they had the authority to question the practice and sometimes saw a need to adapt to changing social circumstances. Further efforts to investigate the trends of FGM/C among the daughters of the survey respondents used in the present study are under way.

The anticipation of reductions in FGM/C detectable in survey data have been driven by several factors. These include combined efforts at the local level, most notably the holistic development program of Tostan that by 2010 culminated in more than 4000 communities participating in public declarations to abandon FGM/C (www.tostan.org, accessed July 2012). Moreover, community-based programs have been supported by a strong national framework to create an “enabling environment” for the abandonment of FGM/C, including developing a detailed national plan of action and implementing legislative reform strategies [1, 7, 26]. Thus, a recent evaluation of the UNFPA-UNICEF Joint Programme on Female Genital Mutilation/Cutting concluded that “Senegal has made concrete progress toward abandonment of FGM/C,” and further speculated that “Senegal could be free from the practice in the near future” ([1]).

When examining trends in FGM/C using nationally-representative survey data, it is important to clearly specify what one should expect to see. Several considerations are salient. First, since data on national prevalence provides information on the proportion of women aged 15–49 who have undergone FGM/C, this aggregate number is unlikely to change dramatically in consecutive surveys implemented 5 years apart; women who are cut will remain cut, and the national prevalence estimate changes only as an increasing proportion of uncut women age into the 15–49 group and cut women age out. A more sensitive indicator of change is to look at the prevalence along age cohorts, as we have done in this study. Second, in doing so, we need to take into account age at cutting, as women’s FGM/C status reflects an event that took place some years in the past [1, 2]. In Senegal, over 60% of girls are cut by age 1, and nearly three quarters of girls are cut before the age of 5 [1]. Thus, when looking at rates of FGM/C in women aged 15–19, we are examining the results of an event that likely took place between 11 and 19 years prior to collection of the survey data. Notably, the youngest cohort of women in the 2010–11 survey were born prior to passage of the law banning FGM/C and at a time where the Tostan program’s work on FGM/C was just beginning to scale up in Senegal [5]. To detect more recent changes, it is possible to examine trends in FGM/C among daughters of the survey respondents. The results of our analysis of daughter data from Senegal DHS are forthcoming. Given the timing of scaled-up local intervention activities and implementation of legal reform efforts, it may not be reasonable to expect to see a dramatic decline in FGM/C risk in the youngest cohort of women in the 2010–11 survey. However, the finding of increased risk of FGM/C in the youngest women in the 2005 and 2010–11 samples is a puzzling finding that we cannot easily explain. Further research will be required to understand this observation, as well as to assess whether FGM/C risk has begun to decline in girls under the age of 15.

## Conclusion

The analysis of two successive survey data from Senegal separately has enabled us to disentangle the complex relationship between FGM/C prevalence and geographical location using an innovative complex Bayesian modelling framework that simultaneously identifies socioeconomic determinants, trends, and the spatial effects that are not explained by these socioeconomic determinants while accounting for the complex sampling scheme. We are able to observe a higher prevalence of FGM/C for younger cohorts (under 20 years of age) for both samples between 2005 and 2010–11 across Senegal and the trends within regions are variable. Some regions managed to reduce the prevalence of FGM/C during the study period while in other regions the prevalence stayed either stagnant or increased. Among factors significantly impacting on FGM/C, we found that younger age of the respondent, urban place of residence, no education, lower household income, being Madingu, Diola and Soninke ethnic group, being of Muslim religion and the region of residence were the most significant factors. The effect of region of residence as a community-level factor, captured by covariate-adjusted geographic estimates is a very significant factor as it mirrors the importance of FGM/C as social norm therefore confirming our initial hypothesis of the social convention theory. The adjusting for individual- and community-level factors has given us a clearer picture of the complex interplay between FGM/C and geography, which needs to be investigated in more detail to see the extent to which this effect is causal or a mere association. After adjusting for individual- and community-level factors, our results show that the prevalence of FGM/C remains high in the young generation of women in Senegal. The different patterns found in the spatial correlation is consistent with the theory of social norms and convention which implies that it would be hard for individuals to jettison the practice of FGM/C without facing harsh social sanctions in their community. For example, the high prevalence region of Matam has the highest risk of FGM/C, while the lowest risk of FGM/C was found in Louga. We note that these spatial patterns may have been influenced by other factors such as place of residence (urban, rural), ethnicity, religion and region. It would be interesting to further examine these factors listed above to see whether the effects are causal or mere association. Also, it was found that ethnicity is a key factor for FGM/C practice. For example, in the 2005 and 2010–11 surveys, Soninke women and Mankingu women were 75.4 and 89.4 times more likely to be cut than Wolof women, respectively (Table 3). This shows that there is an ethnic attachment to the practice of FGM/C. Efforts should therefore target women from such ethnic decent. The unanticipated result on the influence of age on FGM/C reveals the need to further examine the trends of FGM/C in younger women, whilst intervention programmes are also targeted at the younger women. Efforts to investigate the trends of FGM/C among the daughters of the survey respondents are under way. Note that the variations in the Odd Ratios reflect the background influence of other factors unadjusted for. For example, the odds of 'no education' decreased as soon as other factors were controlled, which shows that without the background influence of other factors, women with 'no education' would have an increased risk of FGM/C. This reinforces our hypothesis that geography matters. In other words, FGM/C is a social norm and above the positive impact of education at the individual level, community factors such as the region of residence play a key role in the prevalence of FGM as shown by the significant results of the spatial covariate adjusted multivariate model.

Finally, given that the majority (60%) of the FGM/C are done by age 1, while 75% of the women were cut by age 5. This reveals the need to step up interventions including relevant legislations to stop the practice of FGM/C especially in infancy. Public health priority in Senegal should target regions still showing high prevalence of FGM/C with culturally sensitive policy intervention including legislation, advocacy, education. Considering the geographical shift of FGM/C prevalence, there is also a need for the design of appropriate new studies using the mixed method of longitudinal quantitative and qualitative research design especially among girls.

## APPENDIX

### BAYESIAN GEO-ADDITIVE REGRESSION MODELS

Spatial analyses of FGM often are confined to using region-specific dummy variables to capture the spatial dimension. Here, we go a step further by exploring regional patterns of FGM/C and, possibly nonlinear, effects of other factors within a simultaneous, coherent regression framework using a geo-additive semi-parametric mixed model. Because the predictor contains usual linear terms, nonlinear effects of metrical covariates and geographic effects in additive form, such models are also called geo-additive models. Kammann and Wand [27] proposed this type of model within an empirical Bayesian approach. Here, we apply a fully Bayesian approach as suggested in Fahrmeir and Lang [19], which is based on Markov priors and uses MCMC techniques for inference and model checking.

Classical linear regression models of the form.

1 $$ {y}_i\kern0.5em =\kern0.5em {w_i}^{\hbox{'}}\gamma \kern0.5em +\kern0.5em {\varepsilon}_i,\kern2em {\varepsilon}_i\kern0.5em \sim \kern0.5em N\;\left(0,{\sigma}^2\right)\kern0.5em , $$

for observations (*y*_*i*_,  *w*_*i*_) ,  *i*  =  1, . … , *n*, on a response variable *y* and a vector *w* of covariates assume that the mean *E* (*y*_*i*_ | *w*_*i*_) can be modeled through a *linear predictor w*_*i*_^'^*γ*. In our application to FGM/C and in many other regression situations, we are facing the following problems: First, for the *continuous covariates* in the data set, the assumption of a strictly linear effect on the response *y* may not be appropriate. In our study, such covariate is the respondent’s age. Generally, it will be difficult to model the possibly nonlinear effect of such covariates through a parametric functional form, which has to be *linear* in the parameters, prior to any data analysis.

Second, in addition to usual covariates, geographical small-area information was given in form of a location variable *s*, indicating the region, department or community where individuals or units in the sample size live or come from. In our study, this geographical information is given by the regions in Senegal. Attempts to include such small-area information using region/department-specific dummy-variables would in our case entail more than 30 dummy-variables for the departments and 9 dummies for the regions and using this approach we would not assess spatial inter-dependence. The latter problem cannot also be resolved through conventional multilevel modeling using uncorrelated random effects [15]. It is reasonable to assume that areas close to each other are more similar than areas far apart, so that spatially correlated random effects are required.

To overcome these difficulties, we replace the strictly linear predictor, *η*_*i*_ = *x*^'^*β* + *w*_*i*_^'^*γ* + *ε*_*i*_with a logit link function with dynamic and spatial effects, Pr(y_*i*_ = 1/*η*_*ι*_) = *e*^*ηι*^/(1 + *e*^*ηι*^), and a geo-additive semi-parametric predictor *μ*_*i*_ = *h*(*η*_*ι*_) *:*

2 $$ {\eta}_{\iota }={f}_1\left({x}_{i1}\right)+\dots +{f}_p\left({x}_{ip}\right)+{f}_{spat}\left({s}_i\right)+w{'}_i\gamma +{\varepsilon}_i $$

where *h(.)* is a known response function with a logit link function, *f*_*1*_, …, *f*_*p*_ are non-linear smoothed effects of the metrical covariates (respondent’s age), and *f*_*spat*_ *(s*_*i*_*)* is the effect of the spatial covariate *s*_*i*_ *∈ {1,...,S}* labelling the region in Senegal. Covariates in *w’*_*i*_ are usual categorical variables such as gender and urban-rural residence. Regression models with predictors as in (2) are sometimes referred to as geo-additive models. The observation model (2) may be extended by including interaction *f(x)w* between a continuous covariate *x* and a binary component of *w*, say, leading to so called varying coefficient models, or by adding a nonlinear interaction *f*_*1,2*_ *(x*_*1*_*, x*_*2*_*)* of two continuous covariates.

In a further step, we may split up the spatial effect fspat into a spatially correlated (structured) and an uncorrelated (unstructured) effect

$$ {f}_{spat}\left({s}_i\right)={f}_{str}\left({s}_i\right)+{f}_{untsr}\left({s}_i\right) $$

A rationale is that a spatial effect is usually a surrogate of many unobserved influences, some of them may obey a strong spatial structure and others may be present only locally. By estimating a structured and an unstructured effect, we aim at separating between the two kinds of factors.

As a side effect, we are able to assess to some extent the amount of spatial dependency in the data by observing which one of the two effects is larger. If the unstructured effect exceeds the structured effect, the spatial dependency is smaller and vice versa. It should be noted that all functions are centred about zero for identification purpose, thus fixed effects parameters automatically include an intercept term γ_*0*_.

In a Bayesian approach unknown functions *f*_*j*_ and parameters *γ* as well as the variance parameter *σ*^*2*^ are considered as random variables and have to be supplemented with appropriate prior assumptions. In the absence of any prior knowledge we assume independent diffuse priors *γ*_*j*_ *∝ const, j = 1,...,r* for the parameters of fixed effects. Another common choice is highly dispersed Gaussian priors.

Several alternatives are available as smoothness priors for the unknown functions *f*_*j*_ *(x*_*j*_*)*, see Fahrmeir and Lang [19], Fahrmeir, Kneib and Lang (2004) [28]. We use Bayesian P(enalized) – Splines, introduced by Eilers and Marx [29] in a frequentist setting. It is assumed that an unknown smooth function *f*_*j*_ *(x*_*j*_*)* can be approximated by a polynomial spline of low degree. The usual choices are cubic splines, which are twice continuously differentiable piecewise cubic polynomials defined for a grid of k equally spaced knot *p* on the relevant interval [*a*, *b*] of the x-axis. Such a spline can be written in terms of a linear combination B-spline basis functions*B*_*m*_(*x*), i.e.

3 $$ f(x)=\sum \limits_{m=1}^l{\beta}_m{B}_m(x) $$

These basis functions have finite support on four neighbouring intervals of the grid, and are zero elsewhere. A comparably small number of knots (usually between 10 and 40) is chosen to ensure enough flexibility in combination with a roughness penalty based on second order difference of adjacent B-spline coefficients to guarantee sufficient smoothness of the fitted curves. In our Bayesian approach this corresponds to second order random walks.

4 $$ {\beta}_m=2{\beta}_{m-1}-{\beta}_{m-2}+{u}_m $$

with Gaussian errors *u*_*m*_~*N*(0, *τ*^2^). The variance parameter *τ*^2^ controls the amount of smoothness, and is also estimated from the data. More details on Bayesian P-Splines can be found in Lang and Brezger [30]. Note that random walks are the special case of B-Splines of degree zero.

We now turn our attention to the spatial effects *f*_*str*_ and *f*_*unstr*_. For the spatially correlated effect *f*_*str*_ (s), s = 1, … S, we choose Markov random field priors common in spatial statistics [31]. These priors reflect spatial neighbourhood relationships. For geographical data one usually assumes that two sites or regions s and r are neighbours if they share a common boundary. Then a spatial extension of random walk models leads to the conditional, spatially autoregressive specification.

5 $$ {f}_{str}(s)\mid {f}_{str}(r),r\ne s\sim N\left(\sum \limits_{r\in {\partial}_s}{f}_{str}(r)/{N}_s,{\tau}^2/{N}_s\right) $$

where *N*_*s*_ is the number of adjacent regions, and *r ∈ ∂*_*s*_ denotes that region r is a neighbour of region s. Thus the (conditional) mean of *f*_*str*_*(s)* is an average of function evaluations *f*_*str*_*(s)* of neighbouring regions. Again the variance τ^*2*^ controls the degree of smoothness.

For a spatially uncorrelated (unstructured) effect *f*_*unstr*_ a common assumption is that the parameters *f*_*unstr*_*(s)* are i.i.d. zero mean Gaussian such that.

6 $$ {f}_{unstr}(s)\mid {\tau^2}_{unstr}\kern0.5em \sim N\left(0,{\tau^2}_{unstr}\right) $$

Variance or smoothness parameters *τ*^2^_*j*_, *j* = 1, … , *p*, *str*, *unstr*, are also considered as unknown and estimated simultaneously with corresponding unknown functions *f*_*j*_. Therefore, hyper-priors are assigned to them in a second stage of the hierarchy by highly dispersed inverse gamma distributions *p*(*τ*^2^_*j*_)~*IG*(*a*_*j*_, *b*_*j*_) with known hyper-parameters *a*_*j*_ and *b*_*j*_. Standard choices for the hyperparameters are a = 1 and b = 0.005 or a = b = 0,001. Jeffrey’s noninformative prior is closer to the later choice, and since practical experience shows that regression parameters depend on the choice of hyperparameters, we have investigated in our application the sensitivity to this choice.

Since some regions in Senegal do not have many neighbours, we have investigated the sensibility of the choice of Markov Random Field (MRF) prior with other priors supported by BayesX such as Gaussian random field (GRF) priors, but the resulting maps from the two priors did not differ much. Therefore, we considered the MRF prior for the spatial effects. For model choice, we routinely used the Deviance Information Criterion (DIC) developed in Spiegelhalter et al. [32] as a measure of fit and model complexity. Before commenting on the substantive results, it is important to point out this model had the best fit after evaluation of the fit criteria using Deviance Information Criteria (DIC).

The model assumes that *f*_*1*_ ()*, f*_*2*_() *and f*_*str*_ are nonlinear effects and spatial effects were the same in all the country. This was confirmed by prior separate analyses of the non-linear effects in other countries, which were found to be remarkably similar. The analysis was carried out using BayesX version 0.9, software for Bayesian inference based on Markov Chain Monte Carlo techniques.

Quite clearly, the methods used here are able to identify more subtle socioeconomic and spatial influences on FGM than reliance on linear models with regional dummy variables. As such, they are useful for diagnostic purposes to identify the need to find additional variables that can account for this spatial structure. Moreover, even if the causes of spatial structures are not fully explained, one can use this spatial information for campaigns to eliminate the practice of FGM and planning purposes, which is gaining increasing importance in policy circles, which attempt to focus the allocation of public resources to the most at high risk population.

Multivariate Bayesian geo-additive regression models were used to evaluate the significance of the POR determined for the fixed effects and spatial effects between prevalence of FGM/C in Senegal. Each factor was looked at separately in unadjusted models using conventional logistic regression models. Next, fully adjusted multivariate Bayesian geo-additive regressions analyses were performed to look again for a statistically significant correlation between these variables, but this time further controlling for any influence from individual (age), ethnicity, education and religious factors. A *P*-value of < 0.05 was considered indicative of a statistically significant difference.

**Advantage of using Bayesian geo-additive regression models compared to conventional logistic regression models**: It is worth mentioning some advantages of our approach over existing ones using, say, logistic models with constant-fixed effects of covariates and fixed (or random) district effects or standard two-level multilevel modelling with unstructured spatial effects. First, the Bayesian model (with spatial element) is able to account for spatial autocorrelation of FGM/C prevalence in Senegal by exploring in unified framework the spatial patterns in the prevalence of FGM/C and possible nonlinear effects of continuous covariates (i.e. age) and the usual fixed-effects covariates (i.e. education, income etc..) within a simultaneous, coherent regression framework using a geo-additive semi-parametric mixed model.

Secondly, with conventional models, it is assumed that the random components at the contextual level (district or region) are mutually independent, even though, in practice, this assumption is not actually implied by these approaches, so correlated random residuals could also be specified (see [33]). Borgoni and Billardi [34] pointed out that the independence assumption has an inherent problem of inconsistency: if the location of the event matters, it makes sense to assume that areas close to each other are more similar than areas that are far apart. Also, the SDHS data are based on a random sample of regions. That is, the structured component introduced here allows us to ‘borrow strength’ from neighbours in order to cope with the sample variation of the region effect and obtain estimates for areas that may have inadequate sample sizes or be un-sampled. We tried several models in order to highlight the differences that can be found by adopting this approach in a spatial context and the possible bias involved with the violation of the independent assumption between aggregated spatial areas. Some of these models have a spatial component and a random component that reflect spatial heterogeneity globally and relative homogeneity among neighbouring regions, while some did not. A failure to take into account the posterior uncertainty in the spatial location (district or region) would overestimate the precision of the prediction of FGM/C prevalence in un-sampled locations. The Bayesian paradigm allows the incorporation of prior knowledge to complement the likelihood of the observed data given the model parameters. For our purpose here, we choose Markov Random Field (MRF) prior. The reason behind this choice is that the dependence introduced via the MRF prior ensures that information from neighbouring regions are shared among neighbours (see, for example, [35]). A major advantage of this is that for sparsely populated regions with neighbours having higher populations, there is a reduced variability of estimates as implied by eq. (5) (for the structured spatial effects), that is, the larger the number of neighbours the smaller the variance. We also performed a sensitivity analysis of the various priors’ assumptions of the spatial effects and the model with spatial effects (with MRF prior) outperformed the other models.

Furthermore, the choice of Markov chain Monte Carlo (MCMC) as the implementation framework of the Bayesian model is due to the flexibility of MCMC for complex Bayesian hierarchical models. MCMC allows sample-based inference for complicated and conditionally specified models in which it is easier to study the posterior surface than the likelihood surface. Also, MCMC techniques allow inference from joint posterior and marginal posterior distributions of any subsets of parameters of interest and easy assessment of the variability of all posterior estimates. It is also easier to obtain an additional knowledge of the shape of the likelihood or posterior surface, such as the mode, skewness and kurtosis.

Finally, the conditional independence structure of the MRF has a computational advantage in that the full conditional distribution of each *f*_*str*_*(*_*s*_*)* (eq. 5) is easily computed with greatly reduced computational cost due to sparseness. From the foregoing we argue that the Bayesian model implemented in MCMC framework is a powerful natural choice when interest is on simultaneously accounting for spatial dependence, nonlinear effects and fixed-effects.

## Footnotes

1 Yoder and Wang [2] describe the evolution of DHS questions designed to determine type of cutting.
